# Feasibility Assessment of Parathyroid Hormone Adsorption by Using Polysaccharide-Based Multilayer Film Systems

**DOI:** 10.3390/polym13132070

**Published:** 2021-06-24

**Authors:** Ruey-Shin Juang, Xing Su, I-Chi Lee

**Affiliations:** 1 Department of Chemical and Materials Engineering, Chang Gung University, Guishan, Taoyuan 33302, Taiwan; rsjuang@mail.cgu.edu.tw (R.-S.J.); cc821108@gmail.com (X.S.); 2 Department of Nephrology, Chang Gung Memorial Hospital, Taoyuan 33305, Taiwan; 3 Department of Biomedical Engineering and Environmental Sciences, National Tsing Hua University, Hsinchu 300044, Taiwan

**Keywords:** chronic kidney disease (CKD), parathyroid hormone (PTH) adsorption, polysaccharide-based multilayer films, blood compatibility

## Abstract

Chronic kidney disease (CKD) is a systemic disorder that combines complex bone and mineral abnormalities. The high level of parathyroid hormone (PTH) in the blood causes irreversible renal dysfunction and cardiovascular disease. Therefore, it is necessary to reduce level of PTH in the blood of patients with uremic state. In this study, chitosan and heparin were chosen to form polysaccharide-based multilayer films based on their antibacterial ability, good biocompatibility and hemocompatibility. In addition, a previous study has revealed that PTH is a heparin/polyanion binding protein because of the similarity of heparin to the cell surface proteoglycans. Subsequently, the surface properties including thickness, surface hydrophobicity and surface charge of a series of multilayer films were analyzed. The PTH adsorption rate of a series of multilayer films was also assessed. The results revealed that the optimizing condition is (CHI/HEP)_2.5_ and 60 min in both PBS only and PBS with the addition of bovine serum albumin, which demonstrated the specific adsorption of PTH on the materials. Furthermore, the hemolysis test also revealed that (CHI/HEP)_2.5_ shows good blood compatibility. It is considered that polysaccharide-based multilayer films may provide an alternative for the surface modification of hemodialysis membranes and equipment.

## 1. Introduction

Chronic kidney disease (CKD) is a global and public problem and generally progresses into chronic renal failure. As the disease progresses, renal replacement therapy with dialysis is necessary. Generally, parathyroid glands are responsible for maintaining extracellular calcium concentrations through the secretion of parathyroid hormone (PTH), an 84-amino acid peptide with a molecular mass of 9.4 kDa, and the secretion is regulated by the plasma concentration of ionized calcium [1]. However, hyperparathyroidism is a disease characterized by excessive secretion of PTH, which is a common finding in CKD patients with renal failure [1,2]. Several studies tried to evaluate the calcium, phosphate and PTH concentrations in dialysis populations and analyzed the associations from abnormal mineral metabolism to cardiovascular disease [3]. Although the mechanism is complex, it is considered that high levels of calcium phosphate product and PTH are associated with an increased relative risk of death and cardiovascular-specific mortality in patients with kidney disease [4]. The development of secondary hyperparathyroidism (SHPT) contributes to abnormalities in calcium, phosphorous and PTH which results in dysregulation of skeletal and cardiovascular mortality [5]. Moderate to severe hyperparathyroidism (PTH concentrations ≥ 600 pg/mL) is especially associated with an increase in the relative risk of death [4]. Therefore, PTH is considered a uremic toxin responsible for many of the abnormalities of the uremic state and bone disease.

Hemodialysis is the most commonly used type of dialysis for CKD patients, enabling them to continue living with end-stage kidney disease for many years. However, hemodialysis membranes are generally optimized for the removal of low-molecular-weight solutes. The properties of dialysis filters affect the efficiency and patients’ lives. PTH has been implicated as a pathogenic factor for abnormalities of the uremic state [6]. Generally, small water soluble molecules with molecular weight < 500 Da, such as urea and sodium, can be removed efficiently and easily by most of the filters. However, PTH is a middle-sized uremic toxin and cannot be removed with conventional dialysis [6,7]. Not only do the traditional dialysis membranes not remove the PTH and other middle- sized toxins, the concentration of PTH tends to accumulate in the body during standard dialysis due to the hemoconcentration effect [8]. Therefore, specific and optimized design for PTH removal is necessary for CKD patients, especially for the patients with hyperparathyroidism disease [9].

Layer-by-layer (LBL) adsorption is based on the alternate deposition of oppositely charged molecules, which provides an easy and convenient surface modification by assembling different biomacromolecules and control over physicochemical properties of the multilayer films [10]. The advantage of this technique includes easy operation, cost effectiveness and high tunability of the final product’s properties, and any material type can be used as a substrate regardless of its shape or size. Among these polyelectrolytes, biologically derived polysaccharides are rapidly gaining attention with enormous potential for new biomedical technologies. Heparin was chosen as the polyanion in this study and is generally used in the clinic due to its blood anticoagulant properties. It has also been used to reduce thrombogenicity and improve the hemocompatibility of blood-contacting biomaterials [11]. Heparin has the highest negative charge density of any known biological polyanion and shows electrostatic interaction with a variety of proteins. The specific interaction has also been used in the purification of heparin-binding proteins in cation-exchange chromatography [12]. It is suggested that the range of cellular activity, such as protein folding, signal transduction and intracellular interaction, is regulated by nonspecific electrostatic interactions between the positive electrostatic surface potential of proteins and negatively charged regions of cellular polyanions. A previous study has revealed that PTH is a heparin/polyanion binding protein because heparin is similar to the cell surface proteoglycans, and it is demonstrated that PTH (1–84) molecules bind per ligand of heparin while increasing the a-helical content of the protein [13]. The hydrophilicity of heparin also prevents adhesion of bacterial cells and nonspecific adhesion. Chitosan was chosen as the polycation in this study because of its demonstrated good antibacterial ability and biocompatibility. It has been shown to prevent bacterial infection in the early stages of surgery and promote wound healing [14,15,16]. The combination of these two polyelectrolytes, heparin and chitosan, has shown enormous potential in a variety of applications including drug delivery, dental repair and vascular treatment [16,17,18,19]. 

The LBL deposition technique can be finely adjusted to produce variation of a series of surface physical properties. In this study, chitosan, a cationic polysaccharide with good biocompatibility and antibacterial properties, and heparin, an anionic biocompatible polysaccharide that possesses strong anticoagulant activity, were chosen to form the LBL multilayer films. The thickness of a series of multilayer films was analyzed to observe the increasing layer-by-layer trend, surface hydrophobicity was determined by contact angle and the surface charge of the end layers was analyzed with an interface potential analyzer. The microenvironment may be tuned by the LBL conjugation of the polysaccharide-based multilayer films, which resulted in the variation of surface physicochemical properties and affected the adsorption efficiency. The parameters that affect the adsorption rate such as layer number, charge of the terminal layer and operation time were optimized. The association of PTH adsorption rate and the physical properties of the surface are discussed. Furthermore, different types of solution for simulating the body and blood environment were compared to determine the optimum parameters for PTH adsorption. The blood compatibility was determined with a hemolysis test. The results of this study may provide an alternative for easy and convenient surface modification of dialysis equipment in the treatment of kidney-related diseases in the future.

## 2. Materials and Methods

### 2.1. Materials

Chitosan (average *M*w ca. 50,000–190,000, 75–85% deacetylation), heparin sodium salt (from porcine intestinal mucosa) and bovine serum albumin (BSA) proteins were purchased from Sigma, Chicago, IL, USA. A BCA assay kit was used for BSA protein quantification and was also purchased from Sigma, USA. Chitosan aqueous solution was adjusted to the desired pH using acetic acid (Avantor Performance Materials, Poland). PTH (1–84) human recombinant was purchased from ProSpec (East Brunswick, NJ, USA), and a PTH ELISA Kit was purchased from Sigma (Chicago, IL, USA). 

### 2.2. Preparation of Polysaccharide-Based Multilayer Films

The preparation of chitosan/heparin multilayer films followed the procedure as described in the literature [20]. Physical deposition of chitosan/heparin multilayer films was performed with the following batch conditions. The coverslip was pretreated by oxygen plasma treatment with a working pressure of about 0.5 Torr in the chamber for 5 min (PDC 32G, Harrick Plasma, New York, NY, USA). Chitosan was prepared at a concentration of 0.01 M in 0.2 M acetic acid solution and then raised to pH 5 with 1 N NaOH. The pretreated glass slides were immersed into 1 mg/mL chitosan solution and placed for 10 min at room temperature and subsequently rinsed with 1 mL of deionized water for 1 min. Then, the glass slides were immersed into 1 mg/mL heparin solution for 10 min and also rinsed with deionized water for 1 min. After repeating this process, multilayer films with certain layer numbers were obtained. Finally, PBS was used to clean and remove uncoupled polysaccharides. The pair materials were signed as (chitosan/heparin) n, where n denotes the number of polyelectrolyte pairs generated by repeating the above steps; n = 0.5 refers to glass–chitosan and n = 1 to chitosan/heparin (n = 1).

### 2.3. Analysis of Thickness, Contact Angle and Surface Electrostatic Potential of Polysaccharide-Based Multilayer Films

First, thickness measurements of series of physical depositing polysaccharide-based multilayer films were conducted with an ellipsometer (alpha SE, J.A. Woollam, Lincoln, NE, USA) with a He-Ne laser (λ) at 632.8 nm and a fixed incident angle of 70°. The thickness of series of polysaccharide-based multilayer films was recorded on 10 different regions of the wafer and were averaged then calculated by the software. Secondly, the surface hydrophobicity of a series of polysaccharide-based multilayer films was measured using a contact angle analyzer (Phoenix mini, Surface Electro Optics, Seoul, Korea). The microsyringe produced 5 μL ultrapure water droplets to determine the contact angle of the surfaces, and each layer was measured in triplicate. Thirdly, the surface charge properties of series of polysaccharide-based multilayer films were determined by zeta potential analysis (ELSZ-2000ZS, Otsuka, Japan). Briefly, series of polysaccharide-based multilayer films were immersed in 10^−3^ M of KCl solution by adjusting the pH value to pH 7.4 and then analyzed using the zeta potential instrument.

### 2.4. Adsorption of PTH on Polysaccharide-Based Multilayer Films 

A series of polysaccharide-based multilayer films were prepared to examine adsorption of PTH in vitro. Adsorption of PTH with an initial concentration of 1000 pg/mL on a series of multilayer films at the following time points were determined by using a PTH EIA kit (Sigma, Chicago, IL, USA): 10, 20, 30, 60, 120 and 180 min. Briefly, 25 µL of sample was collected at each time point and added to the 96-well microplate coated with secondary antibody, to which 50 µL Biotinylated PTH and 50 µL HRP-streptavidin solution was added. The sample was incubated for 2.5 h at room temperature with gentle shaking. Subsequently, the solution was discarded and washed 4 times with PBS solution. TMB regent (100 µL) was added to the solution, which was incubated for 30 min at room temperature in the dark with gentle shaking. Eventually, 50 µL of Stop Solution was directly added to each well, and measurements were taken at an absorbance of 450 nm in a multimode microplate reader (BioTek Instruments, Winooski, VT, USA). 

### 2.5. BSA Adsorption

In order to determine the specificity of PTH adsorption on polysaccharide-based multilayer films, protein adsorption was evaluated using quantitative protein analysis. A solution of 20 µg/mL BSA in 1000 pg/mL PTH was prepared for the protein adsorption test. The multilayer film samples were then incubated with the solution for 60 min at room temperature. The BSA adsorption on a series of polysaccharide-based multilayer films was quantified using the Micro BCA™ Protein Assay Kit (Thermo Scientific, Pierce Protein Research Products, Rockford, IL, USA). Supernatant (0.1 mL) was collected from the samples and mixed with 2 mL of bicinchoninic acid solution, and the amount of adsorbed protein was calculated. 

### 2.6. Hemolysis Test

Hemolysis rate was determined by incubating the samples in diluted blood containing normal sodium chloride saline water and fresh anticoagulant blood in a 9:1 ratio at 37 °C for 1 h. For positive and negative controls, 200 µL anticoagulant in 10 mL distilled water and 200 µL anticoagulant in normal saline water were prepared, respectively. After centrifugation at 1000× *g* for 5 min, the absorbance of the supernatant at 541 nm was recorded. The hemolysis rate was calculated by the following equation: hemolysis rate (%) = (S − N)/(P − N) × 100%, in which S, P and N are the absorbances of the sample, positive control and negative control, respectively.

### 2.7. Statistical Analysis

The data are presented as the mean ± standard deviation (SD) from 3 to 6 independent specimens. 

## 3. Results and Discussion

### 3.1. Preparation and Characterization of Polysaccharide-Based Multilayer Films 

**Scheme 1:**
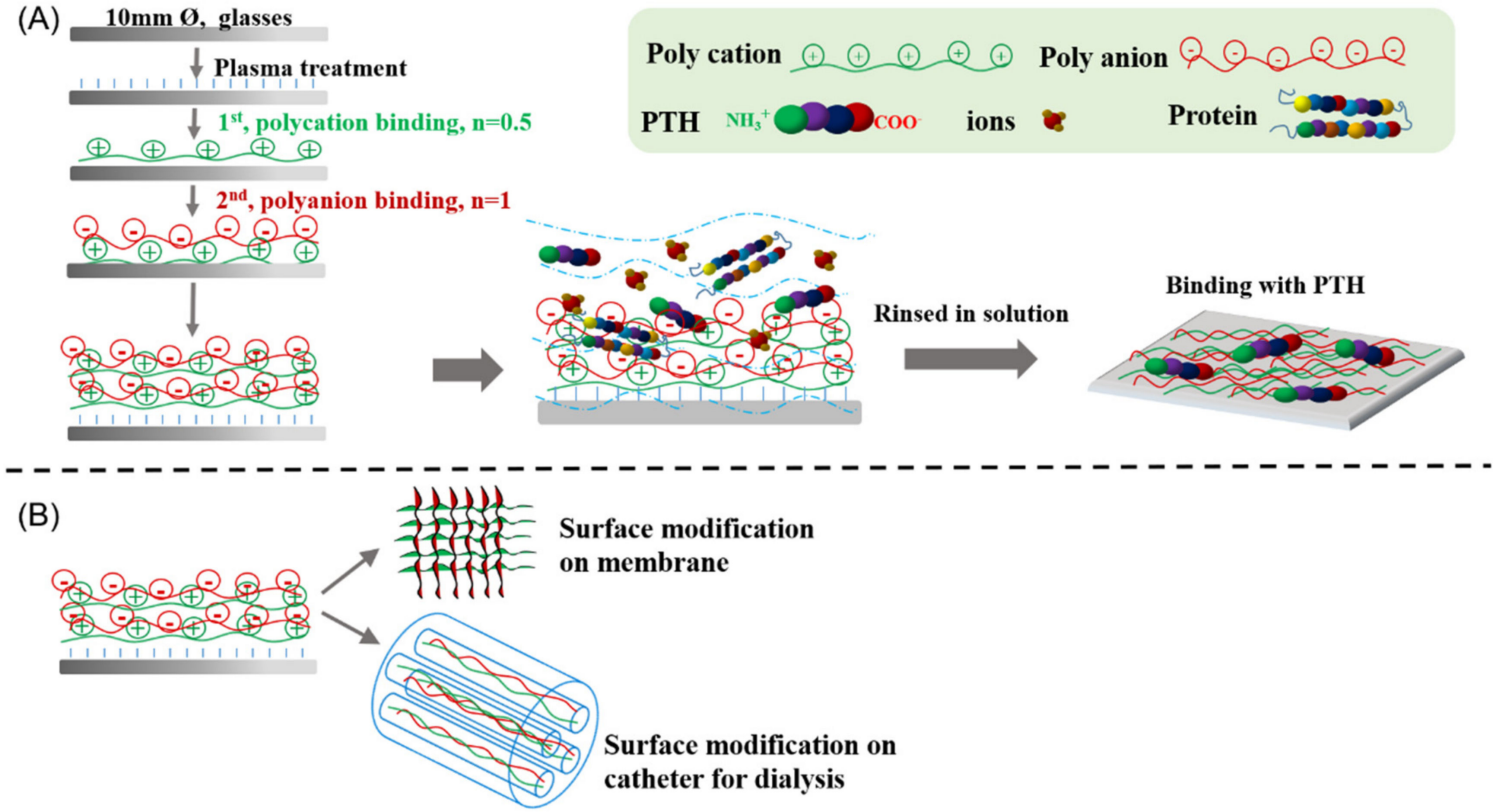
(**A**) Schematic illustrations of layer-by-layer polyelectrolytes adsorbed on the cover glass and PTH adsorption in the environment with other proteins such as BSA. (**B**) Schematic illustrations of surface modification strategies on membrane and hemodialysis catheter. The illustrations are not to scale.

**Figure 1:**
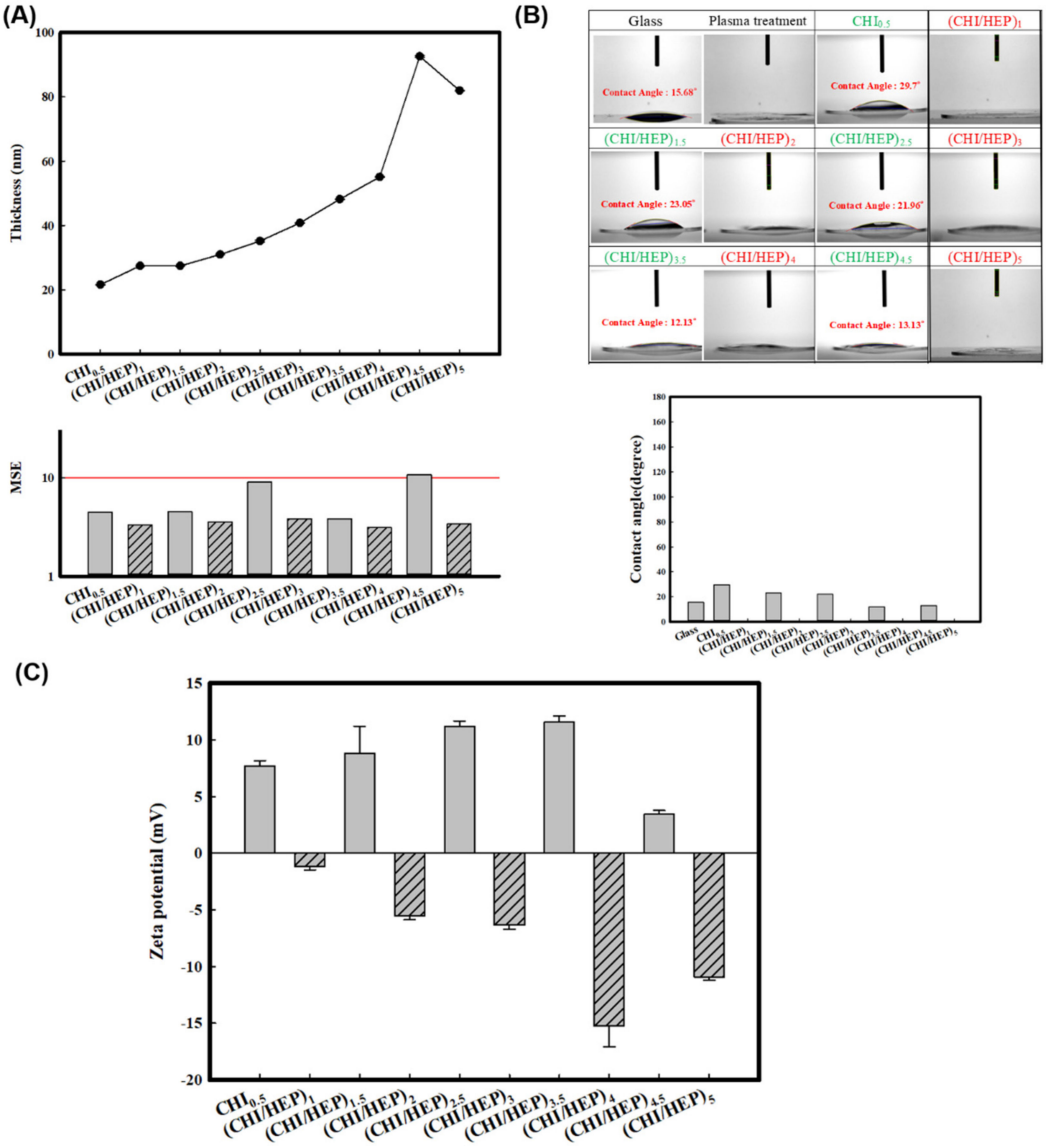
Physical properties of series of polysaccharide-based multilayer films. (**A**) Thickness (**B**) contact angle measurement (**C**) zeta potential analysis.

**Figure 2:**
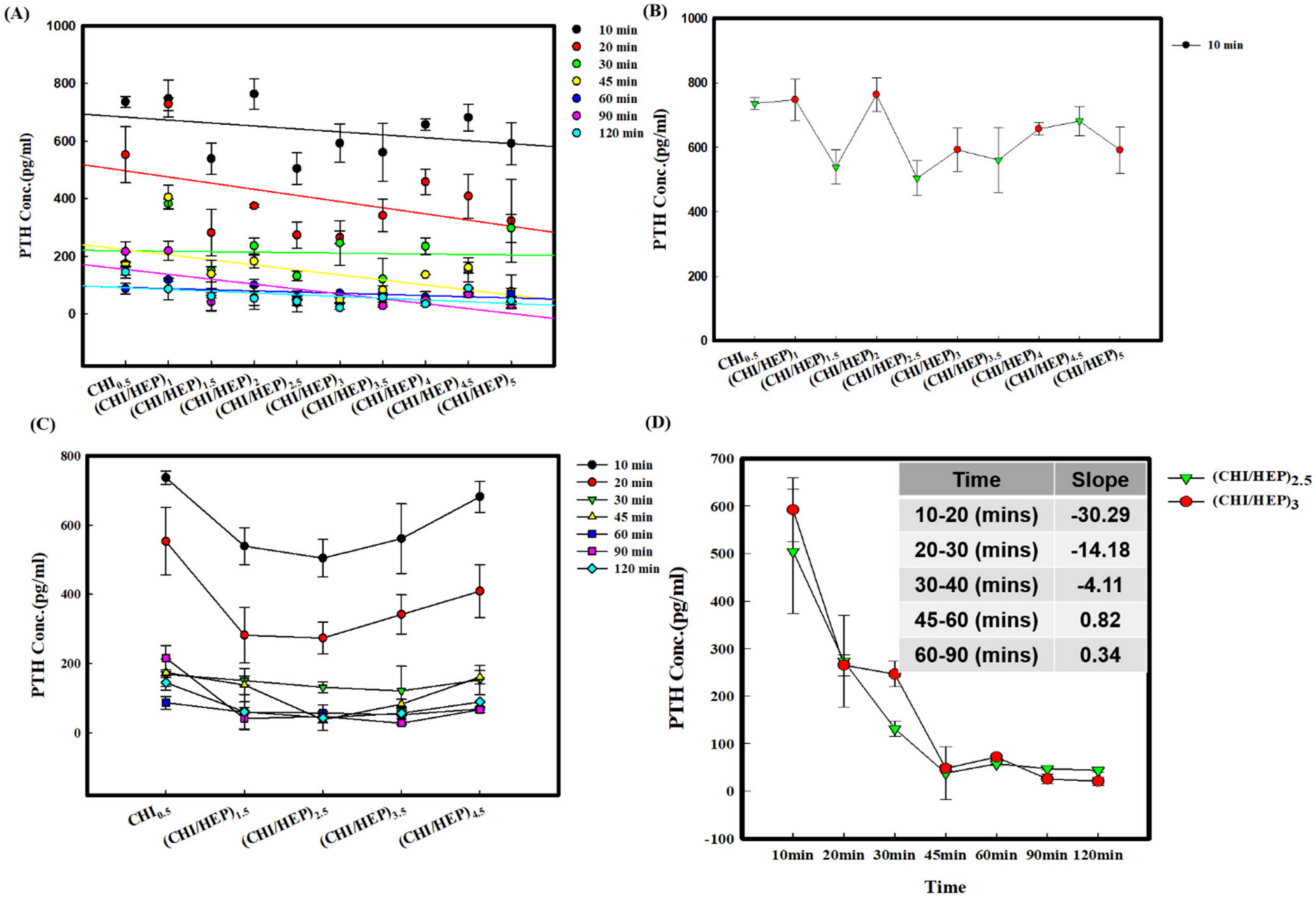
PTH adsorption on a series of polysaccharide-based multilayer films. (**A**) Residual PTH concentration in the solution after different incubation times. (**B**) Residual PTH concentration in the solution after 10 min of incubation. (**C**) Residual PTH concentration in the solution after different incubation times on the terminal layer with positive charge. (**D**) Residual PTH concentration in the solution at different incubation times on (chitosan/heparin)_2.5_ and (chitosan/heparin)_3_. The slopes between each pair of time points on (chitosan/heparin)_2.5_ were analyzed.

The PEM films were prepared with chitosan adsorption on a glass coverslip with plasma treatment, forming a physically coated chitosan film, followed by heparin adsorption generating (chitosan/heparin)_1_ as shown in Scheme 1A. In this study, the films were prepared in up to 5 cycles, with 10 types of samples in total. Physical properties including thickness, surface hydrophobicity and surface zeta potential of series of chitosan/heparin multilayer films were determined as shown in Figure 1. Ellipsometry was utilized to measure the film thickness for all samples. It is shown that the film thickness increased with the number of layers, where 20 nm of chitosan was the thinnest and roughly 90 nm of (chitosan/heparin)_4_._5_ was the thickest. As shown in Figure 2A, the thickness of the 4.5 layer is increased abnormally, and the mean squared error (MSE) is largest one and over the 10, indicating significant difference of this data in comparison with other layers, and the curve should be corrected. Therefore, it is considered that the thicknesses of chitosan/heparin multilayer films are increased as the layer increases. The data confirmed the successful LBL deposition and also demonstrated that the film thickness was controlled by the layer number with the order of 10 to 100 nm.

In order to determine the surface hydrophobicity variation of series of polysaccharide-based multilayer films, the wettability of series of chitosan/heparin films in terms of the deposition numbers was characterized with a contact angle goniometer as shown in Figure 1B. A bare coverslip with a contact angle of θ = 15.68° was used, and the contact angle decreased by θ < 5° after O_2_ plasma treatment. After deposition with chitosan, the contact angle was increased to θ = 29.7° and θ < 5° after deposition with heparin. It was revealed that the multilayer films with a terminal layer of heparin are superhydrophilic with a contact angle of θ < 5°. Generally, the contact angles increased with chitosan as the terminal layer and decreased with heparin as the terminal layer, which is consistent with previous literature [18]. The contact angle slightly decreased as layer increased, which demonstrated that all of the surfaces are hydrophilic and reflect the antifouling characteristics of the multilayer films [18,19]. 

Zeta potential analysis was also performed to monitor the charge accumulation of the surfaces. Figure 1C reveals that all multilayer films with chitosan as the terminal layer exhibited positive zeta potential, and all heparin-ending multilayer films showed negative charge. It was demonstrated that the net surface charge was identified by the terminal layers, indicating charge overcompensation. For both chitosan- and heparin-ending layers, the absolute values of zeta potentials increased as the layer increased to the optimal value and then decreased after layer 4. Combined with the thickness measurement data, this indicates that the stability slightly decreased after deposition with more than 4 layers and affected the accuracy of the measurement of zeta potential and thickness. 

### 3.2. Adsorption of PTH on a Series of Polysaccharide-Based Multilayer Films after Different Incubation Times

Figure 2 shows the PTH adsorption on a series of polysaccharide-based multilayer films in PBS solution. As shown in Figure 2A, it was revealed that the residual PTH concentration in the solution decreased as the incubation time increased on all of the multilayer films. In order to identify the charge effect clearly, Figure 2B shows the residual PTH concentration in the solution after only 10 min of incubation. It was demonstrated that the residual PTH concentration in the solution on the chitosan-ending layer was lower than that on the heparin-ending layer. Furthermore, Figure 2C compares all of the PTH adsorption effects on the films with positively charged terminal layers after different incubation times and the trend of the U-shaped curve, which indicates that the residual PTH concentration in the solution on low and high layers is higher than that on middle layers. It is shown that residual PTH concentration in the solution is the lowest on the (chitosan/heparin)_2.5_ and (chitosan/heparin)_3_ multilayer films. Figure 2D shows that the trends of PTH adsorption on (chitosan/heparin)_2.5_ and (chitosan/heparin)_3_ are similar, and the slopes between each pair of time points reveal that the slope flattened after 60 min of incubation, which demonstrated that the optimized parameters are (chitosan/heparin)_2.5_ with 60 min of incubation. 

**Figure 3:**
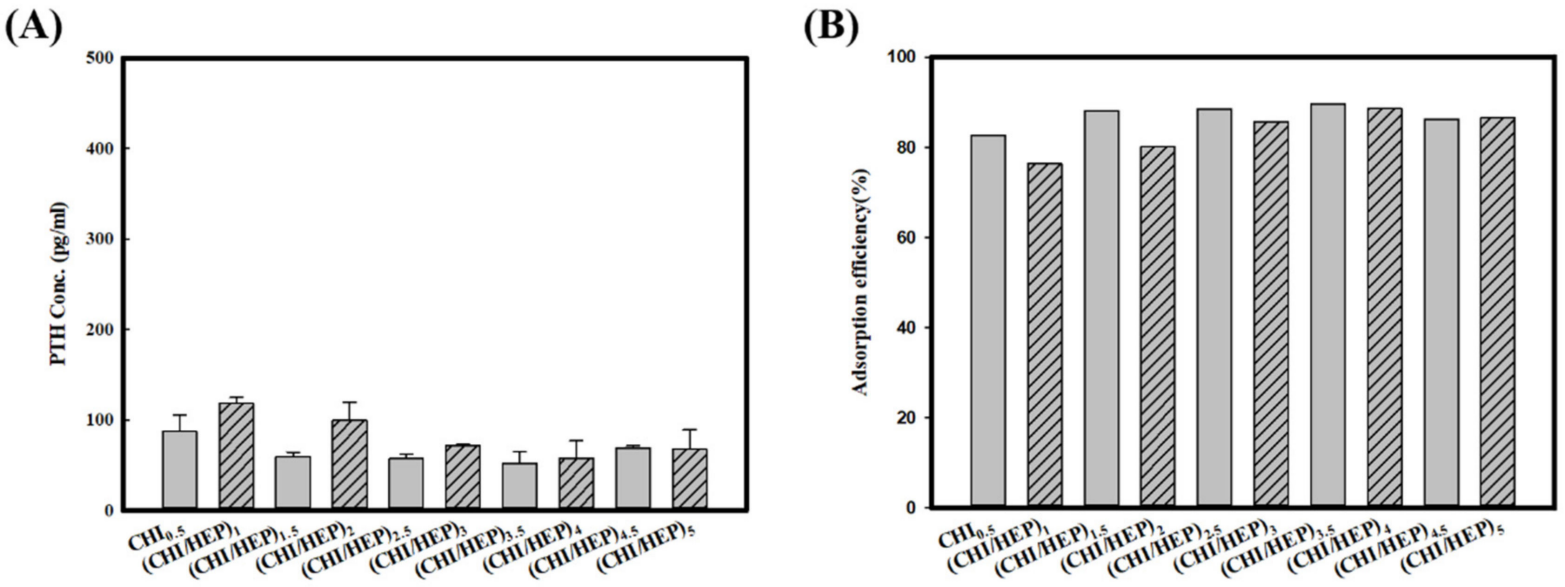
(**A**) Residual PTH concentration in the solution on a series of polysaccharide-based multilayer films after 60 min of incubation. (**B**) Adsorption efficiency of PTH adsorption on a series of polysaccharide-based multilayer films after 60 min of incubation.

It is considered that the incubation time may provide an alternative surface modification strategy for hemodialysis catheters, as shown in Scheme 1B. Figure 3A and Figure S1 show that the trend of residual PTH concentration in water and PBS solution on a series of polysaccharide-based multilayer films after 60 min of incubation were similar. Figure 3B shows the adsorption efficiency of PTH adsorption in PBS solution on a series of polysaccharide-based multilayer films after 60 min of incubation. It was revealed that the (chitosan/heparin)_2.5_ multilayer films presented the highest adsorption efficiency of 88%, and the adsorption efficiency on multilayer films with layer number from 2.5 to 4 was higher than 80%, which demonstrated the high PTH adsorption efficiency on the series of chitosan/heparin multilayer films. A previous study has revealed that moderate to severe hyperparathyroidism (PTH concentrations ≥ 600 pg/mL) is associated with an increase in the relative risk of death [4], and it is suggested that decreasing PTH concentration to < 200pg/mL in the blood may decrease risk. In this study, residual PTH concentration in the solution on a series of polysaccharide-based multilayer films after 60 min of incubation was lower than 200 pg/mL. 

Besides the polysaccharide-based multilayer films, the PTH adsorption of polypeptide-based multilayer films was also analyzed and compared as shown in Figures S2 and S3. It is shown that the adsorption trends are similar, and the optimized incubation time is also 60 min. Based on the following considerations including cost advantage, antibacterial ability and better hemocompatibility, the polysaccharide-based multilayer films were chosen in this study. 

### 3.3. Adsorption of PTH on a Series of Polysaccharide-Based Multilayer Films in the Solution with BSA

**Figure 4:**
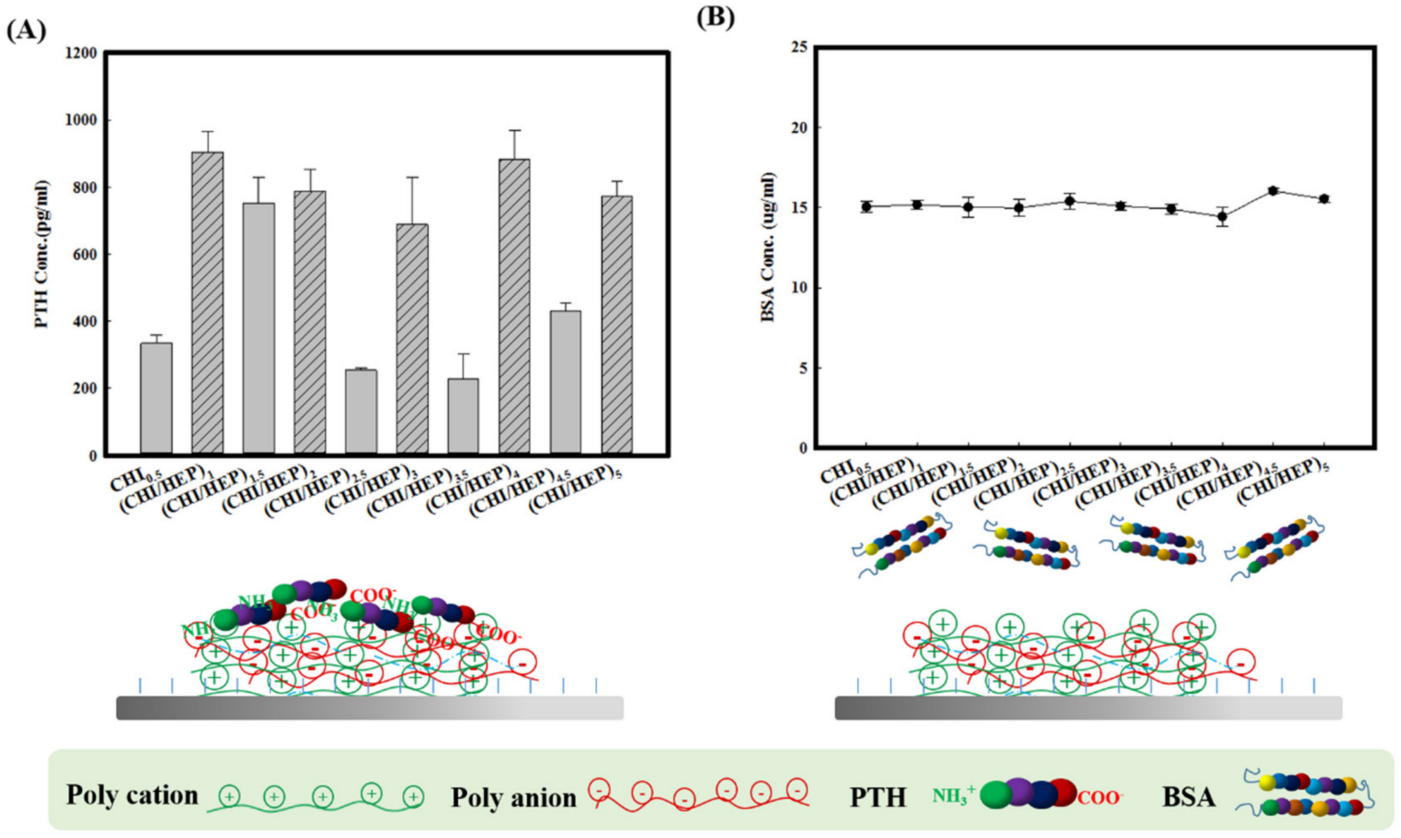
(**A**) Residual PTH concentration on a series of polysaccharide-based multilayer films after 60 min of incubation in the solution with BSA. (**B**) BSA concentration of the solution.

Generally, the fouling process starts from protein adsorption when biomaterials contact with blood or body fluid. The adsorption of PTH in the presence of BSA on polysaccharide-based multilayer films in PBS was investigated because BSA was identified as one of the most important types of adsorbed proteins in blood–material interaction. Jin et al. revealed that polydopamine coated polyurethane film with heparin and carboxymethyl chitosan modification showed a significant decrease of protein adsorption and enhancement of hemocompatibility and antibacterial properties in comparison with polyurethane film [19,21]. Heparin/chitosan complex materials and modification have been widely evidenced to increase hydrophilicity, decrease BSA and fibrinogen adsorption and enhance blood compatibility [22]. In order to model the solution of hemolysis, the PTH adsorption effect in the solution with BSA was investigated, and the BSA quantitative protein analysis was conducted with the BCA kit. Figure 4A shows the residual PTH concentration on a series of polysaccharide-based multilayer films after 60 min of incubation in the solution with BSA. It was revealed that all of the chitosan-ending multilayer films exhibited a better adsorption effect than heparin-ending multilayer films, especially on (chitosan/heparin)_2.5_ multilayer films. In addition, residual PTH concentration in the solution with BSA was higher than that of the environment without BSA. However, as shown in Figure 4B, it was revealed that the BSA concentration in the solution was almost maintained at 15 μg/mL on all of the polysaccharide-based multilayer films, which means the BSA adsorption was not obvious, and it is suggested that the BSA adsorption was not influenced by the zeta potential of the surface and layer numbers. Therefore, it is suggested that the heparin-containing film was resistant to BSA adsorption. Accordingly, the reason for the residual PTH concentration in the solution with increasing BSA is that the degradation rate of PTH was reduced in the solution of BSA and resulted in increased residual PTH concentration in the solution. Thus, the results also revealed that the (chitosan/heparin)_2.5_ multilayer film displayed the best PTH adsorption efficiency, especially in the biomimetic model with BSA. Previous literature has compared the morphology and roughness of a titanium surface after alkali treatment with chitosan and heparin modification. It was demonstrated that the surface of titanium after alkali treatment showed a nanoporous network structure. However, no obvious differences could be found among the different layers of conjugation of heparin and chitosan [23]. The adsorbed masses of PTH on the (chitosan/heparin)_2.5_ multilayer film was higher than that of other multilayer films, and this might be attributed to the hydrophilicity, positive charge of the surface and optimized rougher surfaces of the (chitosan/heparin)_2.5_ multilayer film. For this reason, the (chitosan/heparin)_2.5_ multilayer film was expected to have improved anti-fouling activities, such as reduced nonspecific protein adsorption and decreased bacterial adhesion [18,19].

### 3.4. Blood Compatibility

**Figure 5:**
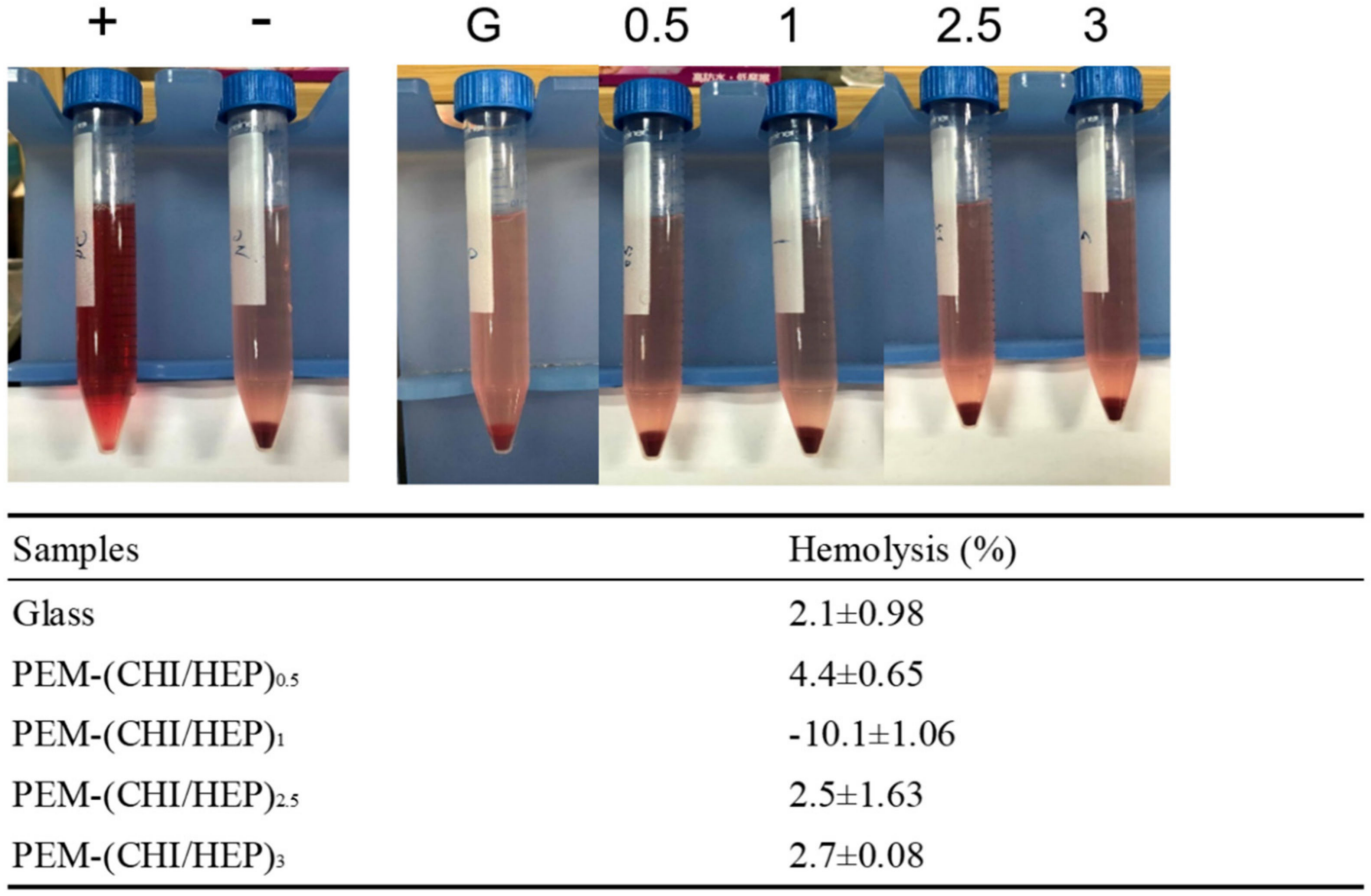
Hemolysis test and hemolysis ratio of series of polysaccharide-based multilayer films. Left column shows positive and negative controls.

In order to characterize blood compatibility of the multilayer films, hemolysis ratio of series of polysaccharide-based multilayer films was determined. As shown in Figure 5, the values for the hemolysis ratios were all lower than 5%, and the ratios of (chitosan/heparin)_2.5_ and (chitosan/heparin)_3_ multilayer film were 2.5% and 2.7%, respectively. No apparent hemolytic activity was observed. The results revealed that the series of heparin-containing materials provided good hemocompatibility even with the chitosan terminal layer, which is consistent with previous studies [22]. Previous literature demonstrated that chitosan/heparin polyelectrolyte complex immobilized onto the surface of polyacrylonitrile membrane can reduce protein adsorption, platelet adhesion and thrombus formation based on heparin immobilization. Therefore, it is considered that heparin-containing polysaccharide-based multilayer films prepared in this study exhibited good blood compatibility, which may provide alternative surface modification strategies for membranes and hemodialysis catheters for PTH adsorption enhancement.

## 4. Conclusions

High levels of PTH in the blood of CKD patients cause irreversible renal dysfunction. This study assessed the feasibility of PTH adsorption by using polysaccharide-based multilayer films. The heparin component provides resistance to BSA adsorption, and chitosan may decrease bacterial adhesion. Chitosan and heparin-containing polysaccharide-based multilayer films showed specific and high adsorption rate of PTH, good biocompatibility, good blood compatibility and low cost, providing an alternative for the surface modification of hemodialysis catheters. 

## Data Availability

The data presented in this study are available on request from the corresponding author.

## Supplementary Materials

The following are available online at https://www.mdpi.com/article/10.3390/polym13132070/s1, Figure S1: Residual PTH concentration in the solution on series of polysaccharide based multilayer films after 60 minutes of incubation. (A) H_2_O solution (B) PBS solution, Figure S2: Residual PTH concentration on series of PLL/PLGA multilayer films in the PBS solution after different incubation times, Figure S3: Residual PTH concentration in the PBS solution at different incubation times on the (PLL/PLGA)_2.5_ and (PLL/PLGA)_3_.
