# Supplementary material for: Feasibility Assessment of Parathyroid Hormone Adsorption by Using Polysaccharide-Based Multilayer Film Systems
Source: Polymers (Basel). 2021 Jun 24;13(13):2070. doi: 10.3390/polym13132070 (PMC8271409; doi:10.3390/polym13132070)
Supplement: Supplementary file 1 [file polymers-13-02070-s001.zip › polymers-1261380-supplementary.pdf]

Feasibility Assessment of Parathyroid Hormone Adsorption by Using Polypeptide and Polysaccharide Multilayer Film Systems

Ruey-Shin Juang [2,3], Xing Su [2], and I-Chi Lee [1,*]

[1] Department of Biomedical Engineering and Environmental Sciences, National Tsing Hua University, Hsinchu, Taiwan

[2] Department of Chemical and Materials Engineering, Chang Gung University, Guishan, Taoyuan 33302, Taiwan

[3] Department of Nephrology, Chang Gung Memorial Hospital, Linkou, Taiwan

* Corresponding author: I-Chi Lee

E-mail: iclee@mx.nthu.edu.tw

Tel: +886-3-5715131 ext 35525

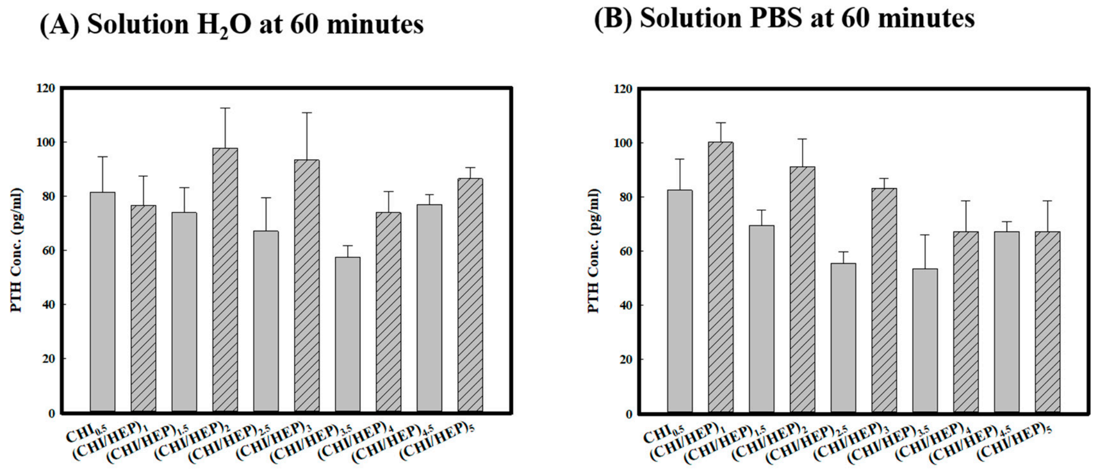

Figure S1. Residual PTH concentration in the solution on series of polysaccharide based multilayer films after 60 minutes of incubation. (A) H_2O solution (B) PBS solution

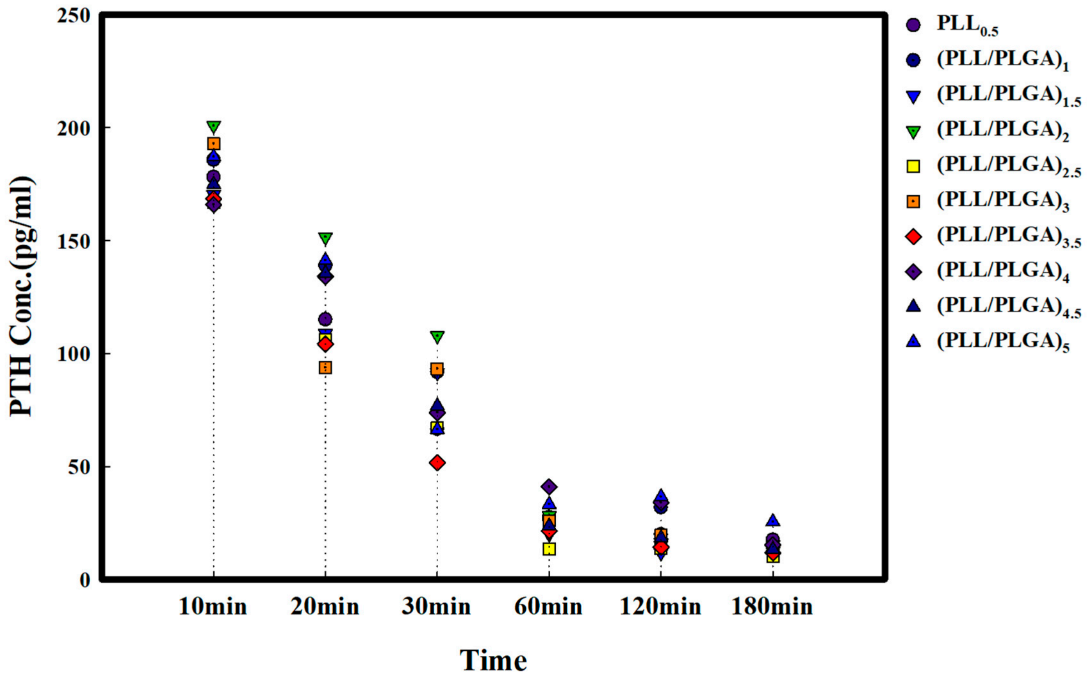

Figure S2. Residual PTH concentration on series of PLL/PLGA multilayer films in the PBS solution after different incubation times.

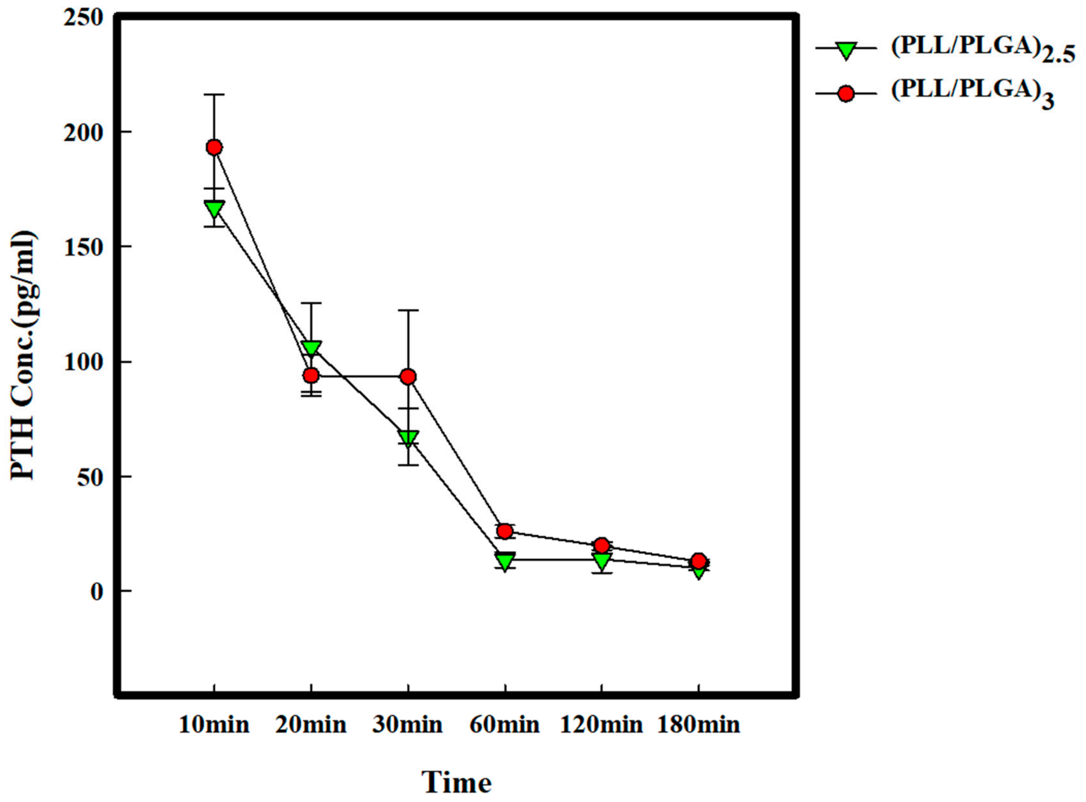

Figure S3. Residual PTH concentration in the PBS solution at different incubation times on the $(PLL/ PLGA)_{2.5}$ and $(PLL/ PLGA)_3$.
